# Pathophysiology and Treatment of Pulmonary Arterial Hypertension

**DOI:** 10.3390/ijms25021166

**Published:** 2024-01-18

**Authors:** Yoshihiro Fukumoto

**Affiliations:** Division of Cardiovascular Medicine, Department of Internal Medicine, Kurume University School of Medicine, Kurume 830-0011, Japan; fukumoto_yoshihiro@kurume-u.ac.jp; Tel.: +81-942-31-7562; Fax: +81-942-33-6509

Pulmonary hypertension (PH) is recognized as a pathophysiological disorder encompassing a wide spectrum of clinical conditions related to various cardiovascular and respiratory diseases [1,2]. The contemporary classification of PH is organized into five clinical groups: Group 1 includes pulmonary arterial hypertension (PAH); Group 2 is associated with left heart disease; Group 3 is related to lung diseases and/or hypoxia; Group 4 involves PH due to pulmonary artery obstructions, including chronic thromboembolic PH (CTEPH); and Group 5 consists of PH with unclear and/or multifactorial mechanisms [2]. Specifically, PAH is identified as a rare and severe condition characterized by remodeling and obstruction of pulmonary microvessels, leading to increased pulmonary vascular resistance, heightened pulmonary arterial pressures, and right ventricular failure [1,2,3]. In cases of hereditary PAH, various germline mutations have been implicated [1,2]. The pathophysiology of PAH is complex, involving mechanisms such as vasoconstriction, vascular remodeling, endothelial dysfunction, inflammation, and thrombosis [1,2]. PAH may arise from several conditions, including connective tissue diseases (CTD-PAH), ref. [4] congenital heart diseases (CHD-PAH), and liver cirrhosis, with these pathological alterations contributing to the progression of Groups 2 and 3 PH [1,2]. In CTEPH, PH may develop from pulmonary artery obstructions due to organized fibrotic clots, potentially coupled with vascular dysfunction across various pulmonary artery sizes [1,2,5,6,7]. Within this pathophysiological framework, several vasoactive mediators, including nitric oxide, prostacyclin, endothelin-1, serotonin, and thromboxane, play roles in the development and subsequent structural remodeling of pulmonary vascular hypertrophy [1,2]. Treatment for CTEPH includes surgical endarterectomy of the affected pulmonary arteries and/or balloon pulmonary angioplasty (BPA) [1,2,8]; however, effective medical therapies targeting the underlying causes of pulmonary arteriopathy in CTEPH and PAH remain underdeveloped.

This Special Issue primarily concentrates on PAH, classified as Group 1, with the objective of elucidating recently identified cellular pathogenesis mechanisms. This insight is anticipated to pave the way for novel and innovative treatment options and strategies. Additionally, it addresses CTEPH, categorized as Group 4, further expanding the scope of this discussion.

Tomaszewski et al. [9] reported that idiopathic PAH patients showed a significantly higher percentage of CD4+CTLA-4+ and CD8+CTLA-4+ T lymphocytes compared to controls and other PAH subtypes. These elevated levels correlate with increased severity of heart failure according to the World Health Organization (WHO) Functional Classification and higher N-terminal pro-B-type natriuretic peptide (NT-proBNP) levels, suggesting their potential as prognostic markers. Specifically, higher percentages of these lymphocytes at diagnosis are associated with increased mortality within five years, indicating their value in predicting disease outcome. While CTLA-4+ cells show promise as noninvasive biomarkers for detecting idiopathic PAH, their role in treatment strategy is yet to be determined. Further research is needed to understand the relationship between T and B lymphocyte subsets and different PAH types [9].

Tornling et al. [10] demonstrated that in the Sugen–Hypoxia rat model, the AT2R agonist C21’s effects on remodeling, hemodynamic alterations, and augmented pulmonary collagen deposition suggest its potential therapeutic role not only in PAH but also in addressing the critical need for treatments in Group 3 pulmonary hypertension associated with interstitial lung disease (ILD). This potential extends to cases of systemic sclerosis and other connective tissue diseases where Group 1 pulmonary hypertension emerges concurrently with lung fibrosis [10].

Further, Peng et al. [11] evaluated the efficacy of three FDA-approved vasodilating drugs in a PAH mouse model (*Egln1^Tie2Cre^* mice) that spontaneously develops severe PAH with stable occlusive pulmonary vascular remodeling and right ventricular failure. While these drugs reduced right ventricular systolic pressure and improved cardiac output, they had limited impact on pulmonary artery function, right ventricular hypertrophy, and pulmonary vascular remodeling, with variable survival benefits. These findings align with clinical trial outcomes in PAH patients. This study underscores the *Egln1^Tie2Cre^* mouse as a representative model for severe PAH, valuable for identifying potential drug targets and preclinical testing of novel therapies [11].

Ryanto et al. [12] reviewed the role of activin/inhibin signaling as a crucial mediator in the development of PAH and its potential as a therapeutic target to enhance patient outcomes. They established the significant involvement of activins and inhibins in PAH pathogenesis, and contend that realizing the full therapeutic potential of targeting activins/inhibins in PAH necessitates extensive further basic and clinical research [12].

Pullamsetti et al. [13] discussed the significance of the PDGFR-CSF1R-c-KIT signaling network in PAH pathogenesis and proposed that inhibiting all three nodes could be a novel therapeutic strategy. They highlighted the potential of seralutinib, a drug under development targeting these pathways. This novel PDGFR/CSF1R/c-KIT inhibitor, delivered via inhalation, is particularly potent against PDGFRβ and CSF1R, with features distinct from imatinib, including low oral bioavailability ideal for inhalation administration. Seralutinib has demonstrated preclinical efficacy in multiple animal models and restored BMPR2 levels in a specific model of PH, indicating its potential for reversing pulmonary vascular remodeling and improving clinical outcomes in PAH. Ongoing research, including techniques like single-cell RNA sequencing, aims to further elucidate seralutinib’s mechanisms and its impact on PAH’s pathological remodeling. Preliminary results from a randomized controlled trial showed seralutinib significantly reduced pulmonary vascular resistance and was well-tolerated, leading to a forthcoming phase 3 study to further assess its efficacy and safety in PAH. This research suggests seralutinib, especially in combination with other therapies, may offer a promising new approach to treating PAH [13].

In this Special Issue, Otani et al. [14] reviewed CTEPH, a condition where an organic thrombus persists in the pulmonary artery despite over three months of anticoagulation therapy, leading to right-sided heart failure and potentially fatal outcomes. CTEPH, characterized by pulmonary artery stenosis and occlusion from organized thrombi, results in progressive pulmonary vascular disease and a poor prognosis if untreated. The standard treatment, PEA, is typically offered in specialized centers and is recommended for operable cases. Recently, BPA and drug therapies, such as riociguat, have shown promising results. The review highlights the complex pathogenesis of CTEPH, involving inflammatory cytokines and coagulation abnormalities, and emphasizes the importance of early detection and intervention to improve prognosis. While PEA remains the curative standard, the increasing success of BPA and drug therapy offers hope for inoperable cases. Future advancements are anticipated to further improve outcomes in CTEPH treatment [14].

Despite advancements of pulmonary vasodilators (with various side effects [15]) over the past two decades, PAH and CTEPH remain serious conditions with significant morbidity and mortality [16]. To date, no treatments directly addressing the underlying causes of pulmonary arteriopathy have been developed. However, new therapeutic targets have emerged, with some progressing to market availability and clinical application. We earnestly hope for continued research into the etiology of PAH and CTEPH, leading to the establishment of fundamental treatments. 
